# *tert*-Butyl 2-{[2,8-bis­(trifluoro­meth­yl)quinolin-4-yl](hy­droxy)meth­yl}piperidine-1-carboxyl­ate

**DOI:** 10.1107/S1600536811047726

**Published:** 2011-11-16

**Authors:** Raoni S. B. Gonçalves, Marcus V. N. de Souza, James L. Wardell, Solange M. S. V. Wardell, Edward R. T. Tiekink

**Affiliations:** a Fundaçaõ Oswaldo Cruz, Instituto de Tecnologia, em Fármacos–Farmanguinhos, R. Sizenando Nabuco, 100, Manguinhos, 21041-250 Rio de Janeiro, RJ, Brazil; b Centro de Desenvolvimento Tecnológico em Saúde (CDTS), Fundação Oswaldo Cruz (FIOCRUZ), Casa Amarela, Campus de Manguinhos, Av. Brasil 4365, 21040-900 Rio de Janeiro, RJ, Brazil; c CHEMSOL, 1 Harcourt Road, Aberdeen AB15 5NY, Scotland; d Department of Chemistry, University of Malaya, 50603 Kuala Lumpur, Malaysia

## Abstract

The title mol­ecule, C_22_H_24_F_6_N_2_O_3_, adopts a folded conformation whereby the carboxyl­ate residue lies over the quinolinyl residue, with the dihedral angle between the carbamate and quinoline planes being 41.64 (7)°. Helical supra­molecular *C*(7) chains sustained by O—H⋯O hydrogen bonds propagating along the *a*-axis direction feature in the crystal packing. The F atoms of one of the CF_3_ groups are disordered over two orientations; the major component has a site occupancy of 0.824 (7).

## Related literature

For background to the anti-mycobacterial activity of mefloquine, see: Gonçalves *et al.* (2010 ▶); Mao *et al.* (2007 ▶); Maguire *et al.* (2006 ▶). For the synthesis, see: Grellepois *et al.* (2005 ▶). For related structural studies, see: Gonçalves *et al.* (2011 ▶); de Souza *et al.* (2011 ▶); Wardell *et al.* (2010 ▶, 2011*a* ▶,*b* ▶); Pitaluga *et al.* (2010 ▶).
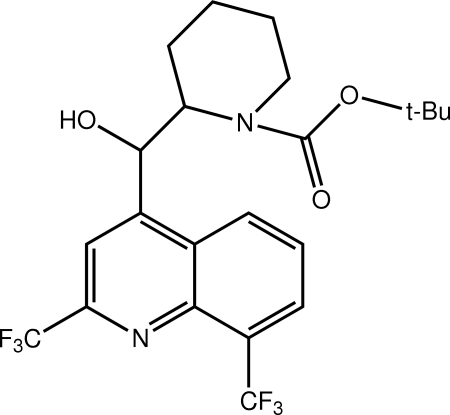


## Experimental

### 

#### Crystal data

C_22_H_24_F_6_N_2_O_3_
*M* *_r_* = 478.43
Orthorhombic, 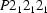

*a* = 9.888 (3) Å
*b* = 10.696 (3) Å
*c* = 21.158 (5) Å
*V* = 2237.7 (10) Å^3^
*Z* = 4
Cu *K*α radiation
μ = 1.12 mm^−1^
*T* = 100 K
0.12 × 0.11 × 0.10 mm

#### Data collection

Rigaku Saturn944+ diffractometer
Absorption correction: multi-scan (*CrystalClear-SM Expert*; Rigaku, 2011 ▶) *T*_min_ = 0.863, *T*_max_ = 1.000
10899 measured reflections
3722 independent reflections
3640 reflections with *I* > 2σ(*I*)
*R*_int_ = 0.037

#### Refinement

*R*[*F* ^2^ > 2σ(*F* ^2^)] = 0.054
*wR*(*F* ^2^) = 0.130
*S* = 1.13
3722 reflections
314 parameters
31 restraints
H atoms treated by a mixture of independent and constrained refinement
Δρ_max_ = 0.37 e Å^−3^
Δρ_min_ = −0.44 e Å^−3^
Absolute structure: Flack (1983 ▶), 1532 Friedel pairs
Flack parameter: −0.08 (13)

### 

Data collection: *CrystalClear-SM Expert* (Rigaku, 2011 ▶); cell refinement: *CrystalClear-SM Expert*; data reduction: *CrystalClear-SM Expert*; program(s) used to solve structure: *SHELXS97* (Sheldrick, 2008 ▶); program(s) used to refine structure: *SHELXL97* (Sheldrick, 2008 ▶); molecular graphics: *ORTEP-3* (Farrugia, 1997 ▶) and *DIAMOND* (Brandenburg, 2006 ▶); software used to prepare material for publication: *publCIF* (Westrip, 2010 ▶).

## Supplementary Material

Crystal structure: contains datablock(s) global, I. DOI: 10.1107/S1600536811047726/hb6499sup1.cif

Structure factors: contains datablock(s) I. DOI: 10.1107/S1600536811047726/hb6499Isup2.hkl

Supplementary material file. DOI: 10.1107/S1600536811047726/hb6499Isup3.cml

Additional supplementary materials:  crystallographic information; 3D view; checkCIF report

## Figures and Tables

**Table 1:** Hydrogen-bond geometry (Å, °)

*D*—H⋯*A*	*D*—H	H⋯*A*	*D*⋯*A*	*D*—H⋯*A*
O1—H1*o*⋯O2^i^	0.85 (3)	1.96 (3)	2.794 (3)	171 (3)

Symmetry code: (i) 
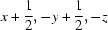
.

**Fig. 1.**
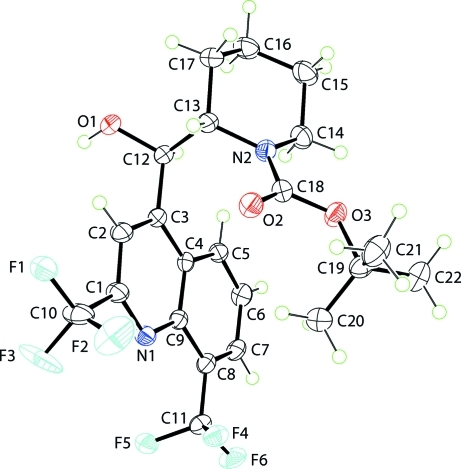
The molecular structure of (I) showing displacement ellipsoids at the 50% probability level.

**Fig. 2.**
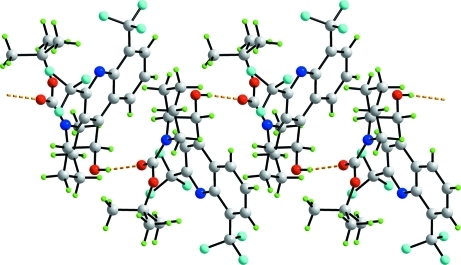
A view of the helical supramolecular chain propagated long the a axis via O—H···O hydrogen bonds (orange dashed lines) in the crystal structure of (I).

## supplementary crystallographic information

### Comment

Mefloquine has been used in the prevention and treatment for malaria in combination with other drugs for some decades (Maguire *et al.*, 2006). More recently, the activity of mefloquine has been investigated against other diseases, for example, as anti-viral and anti-tubercular agents (Mao *et al.*, 2007). In continuation of our structural and biological studies on mefloquine derivatives (Gonçalves *et al.*, 2010, 2011; de Souza *et al.* 2011; Wardell, *et al.*, 2010; 2011*a*; 2011*b*; Pitaluga *et al.*, 2010), we now report the crystal and molecular structure of the title compound, *tert*-butyl 2-[[2,8-bis(trifluoromethyl)-4-quinolinyl](hydroxy)methyl]tetrahydro-1(2*H*)-pyridine carboxylate, (I).

In the molecule of (I), Fig. 1, the hydroxyl group lies to one side of the plane through the quinolinyl residue and the substituted piperidine ring to other with the carboxylate ester folded over to lie over the quinolinyl plane. However, the two residues are non-parallel with the dihedral angle between the quinolinyl and carbamate planes being 41.64 (7) °. The piperidine ring has a conformation close to a chair form. Mefloquine used as a reagent was a racemate. However, the reported crystal structure is of one form of a racemic mixture as the molecules spontaneously resolved during crystallization. The sum of the angles at the site of substitution, the trisubstituted N2 atom, is 360° indicating a planar geometry, and hence an achiral centre. In (I), the configurations at the C12 and C13 positions are *R* and *S*, respectively. The most prominent intermolecular interactions in the crystal structure are O—H···O hydrogen bonds that lead to helical supramolecular chains along the *a* axis, Fig. 1 and Table 1.

### Experimental

The compound was prepared by a published procedure (Grellepois *et al.*, 2005) from Boc_2_O and mefloquine in the presence of Et_3_N. Colourless chips of (I) were grown from an EtOH solution, *M*.pt. 419–420 K.

### Refinement

The C-bound H atoms were geometrically placed (C—H = 0.95–1.00 Å) and refined as riding with *U_iso_*(H) = 1.2–1.5*U_eq_*(C). The O—H H atom was located in a difference and refined with O—H = 0.84±0.01 Å, and with *U_iso_*(H) = 1.5*U_eq_*(O). One of the CF_3_ groups was found to be disordered. This was resolved over two positions for each F atom. The pairs of F atom had common anisotropic displacement parameters. The major component of the disordered F atoms had a site occupancy = 0.824 (7).

### Crystal data

**Table:**

C_22_H_24_F_6_N_2_O_3_	*F*(000) = 992
*M**_r_* = 478.43	*D*_x_ = 1.420 Mg m^−^^3^
Orthorhombic, *P*2_1_2_1_2_1_	Cu *K*α radiation, λ = 1.54187 Å
Hall symbol: P 2ac 2ab	Cell parameters from 2676 reflections
*a* = 9.888 (3) Å	θ = 21.7–66.3°
*b* = 10.696 (3) Å	µ = 1.12 mm^−^^1^
*c* = 21.158 (5) Å	*T* = 100 K
*V* = 2237.7 (10) Å^3^	Chip, colourless
*Z* = 4	0.12 × 0.11 × 0.10 mm

### Data collection

**Table:**

Rigaku Saturn944+ diffractometer	3722 independent reflections
Radiation source: fine-focus sealed tube	3640 reflections with *I* > 2σ(*I*)
confocal	*R*_int_ = 0.037
Detector resolution: 22.2222 pixels mm^-1^	θ_max_ = 66.5°, θ_min_ = 4.9°
profile data from ω–scans	*h* = −11→11
Absorption correction: multi-scan (*CrystalClear-SM Expert*; Rigaku, 2011)	*k* = −11→12
*T*_min_ = 0.863, *T*_max_ = 1.000	*l* = −25→24
10899 measured reflections	

### Refinement

**Table:**

Refinement on *F*^2^	Secondary atom site location: difference Fourier map
Least-squares matrix: full	Hydrogen site location: inferred from neighbouring sites
*R*[*F*^2^ > 2σ(*F*^2^)] = 0.054	H atoms treated by a mixture of independent and constrained refinement
*wR*(*F*^2^) = 0.130	*w* = 1/[σ^2^(*F*_o_^2^) + (0.0998*P*)^2^ + 0.2114*P*] where *P* = (*F*_o_^2^ + 2*F*_c_^2^)/3
*S* = 1.13	(Δ/σ)_max_ < 0.001
3722 reflections	Δρ_max_ = 0.37 e Å^−^^3^
314 parameters	Δρ_min_ = −0.44 e Å^−^^3^
31 restraints	Absolute structure: Flack (1983), 1532 Friedel pairs
Primary atom site location: structure-invariant direct methods	Flack parameter: −0.08 (13)

### Special details

**Table:**

Geometry. All s.u.'s (except the s.u. in the dihedral angle between two l.s. planes) are estimated using the full covariance matrix. The cell s.u.'s are taken into account individually in the estimation of s.u.'s in distances, angles and torsion angles; correlations between s.u.'s in cell parameters are only used when they are defined by crystal symmetry. An approximate (isotropic) treatment of cell s.u.'s is used for estimating s.u.'s involving l.s. planes.
Refinement. Refinement of *F*^2^ against ALL reflections. The weighted *R*-factor *wR* and goodness of fit *S* are based on *F*^2^, conventional *R*-factors *R* are based on *F*, with *F* set to zero for negative *F*^2^. The threshold expression of *F*^2^ > 2σ(*F*^2^) is used only for calculating *R*-factors(gt) *etc*. and is not relevant to the choice of reflections for refinement. *R*-factors based on *F*^2^ are statistically about twice as large as those based on *F*, and *R*- factors based on ALL data will be even larger.

### Fractional atomic coordinates and isotropic or equivalent isotropic displacement parameters (Å^2^)

**Table:**

	*x*	*y*	*z*	*U*_iso_*/*U*_eq_	Occ. (<1)
C10	0.2018 (3)	−0.0161 (2)	0.10080 (11)	0.0390 (6)	0.824 (7)
F1	0.1438 (3)	−0.0585 (4)	0.04787 (14)	0.0360 (7)	0.824 (7)
F2	0.0959 (3)	−0.0005 (3)	0.14211 (13)	0.0639 (10)	0.824 (7)
F3	0.2775 (3)	−0.1073 (2)	0.1234 (2)	0.0713 (10)	0.824 (7)
C10'	0.2018 (3)	−0.0161 (2)	0.10080 (11)	0.0390 (6)	0.176 (7)
F1'	0.1146 (16)	−0.050 (2)	0.0558 (8)	0.0360 (7)	0.176 (7)
F2'	0.1618 (18)	−0.0399 (15)	0.1586 (3)	0.0639 (10)	0.176 (7)
F3'	0.3022 (11)	−0.1023 (11)	0.0948 (8)	0.0713 (10)	0.176 (7)
F4	0.36443 (14)	0.19344 (13)	0.26991 (6)	0.0295 (3)	
F5	0.55029 (14)	0.10401 (13)	0.24085 (6)	0.0304 (3)	
F6	0.55644 (16)	0.27288 (15)	0.29575 (6)	0.0368 (4)	
O1	0.31994 (19)	0.27116 (16)	−0.08675 (7)	0.0322 (4)	
H1o	0.387 (3)	0.222 (3)	−0.0866 (17)	0.058*	
O2	0.05287 (17)	0.37519 (15)	0.07601 (7)	0.0294 (4)	
O3	0.11139 (19)	0.56561 (16)	0.11565 (8)	0.0303 (4)	
N1	0.3449 (2)	0.14152 (17)	0.14112 (8)	0.0235 (4)	
N2	0.1754 (2)	0.51397 (18)	0.01744 (8)	0.0257 (4)	
C1	0.2766 (2)	0.1044 (2)	0.09080 (11)	0.0265 (5)	
C2	0.2698 (2)	0.1688 (2)	0.03333 (11)	0.0270 (5)	
H2	0.2194	0.1357	−0.0011	0.032*	
C3	0.3375 (2)	0.2811 (2)	0.02730 (9)	0.0219 (5)	
C4	0.4146 (2)	0.3242 (2)	0.07962 (10)	0.0199 (4)	
C5	0.4941 (2)	0.4353 (2)	0.07868 (10)	0.0226 (4)	
H5	0.4952	0.4858	0.0417	0.027*	
C6	0.5686 (2)	0.4702 (2)	0.13000 (11)	0.0273 (5)	
H6	0.6216	0.5442	0.1282	0.033*	
C7	0.5678 (2)	0.3978 (2)	0.18567 (10)	0.0250 (5)	
H7	0.6201	0.4232	0.2211	0.030*	
C8	0.4923 (2)	0.2916 (2)	0.18890 (9)	0.0228 (5)	
C9	0.4150 (2)	0.2504 (2)	0.13550 (9)	0.0202 (4)	
C11	0.4905 (2)	0.2163 (2)	0.24812 (10)	0.0249 (5)	
C12	0.3230 (2)	0.3538 (2)	−0.03392 (10)	0.0239 (5)	
H12	0.3999	0.4139	−0.0385	0.029*	
C13	0.1870 (2)	0.4255 (2)	−0.03579 (10)	0.0251 (5)	
H13	0.1130	0.3625	−0.0311	0.030*	
C14	0.2429 (3)	0.6364 (2)	0.01233 (11)	0.0313 (5)	
H14A	0.3419	0.6247	0.0165	0.038*	
H14B	0.2125	0.6908	0.0474	0.038*	
C15	0.2123 (3)	0.7003 (2)	−0.05050 (12)	0.0374 (6)	
H15A	0.1154	0.7233	−0.0522	0.045*	
H15B	0.2663	0.7779	−0.0541	0.045*	
C16	0.2459 (3)	0.6138 (2)	−0.10499 (12)	0.0363 (6)	
H16A	0.3438	0.5946	−0.1047	0.044*	
H16B	0.2239	0.6553	−0.1455	0.044*	
C17	0.1649 (3)	0.4927 (2)	−0.09901 (11)	0.0314 (5)	
H17A	0.1908	0.4358	−0.1339	0.038*	
H17B	0.0675	0.5121	−0.1037	0.038*	
C18	0.1084 (2)	0.4769 (2)	0.07035 (10)	0.0257 (5)	
C19	0.0610 (2)	0.5418 (2)	0.18031 (10)	0.0281 (5)	
C20	0.1404 (3)	0.4359 (3)	0.20999 (12)	0.0363 (6)	
H20A	0.2374	0.4523	0.2055	0.054*	
H20B	0.1177	0.3572	0.1887	0.054*	
H20C	0.1173	0.4296	0.2549	0.054*	
C21	−0.0903 (3)	0.5172 (3)	0.17923 (13)	0.0397 (6)	
H21A	−0.1256	0.5193	0.2225	0.060*	
H21B	−0.1075	0.4348	0.1606	0.060*	
H21C	−0.1353	0.5816	0.1539	0.060*	
C22	0.0909 (3)	0.6644 (3)	0.21311 (13)	0.0385 (6)	
H22A	0.1879	0.6824	0.2103	0.058*	
H22B	0.0643	0.6586	0.2576	0.058*	
H22C	0.0398	0.7317	0.1926	0.058*	

### Atomic displacement parameters (Å^2^)

**Table:**

	*U*^11^	*U*^22^	*U*^33^	*U*^12^	*U*^13^	*U*^23^
C10	0.0500 (15)	0.0332 (13)	0.0339 (12)	−0.0136 (12)	−0.0226 (12)	0.0086 (11)
F1	0.0424 (15)	0.0259 (10)	0.0397 (11)	−0.0088 (13)	−0.0189 (12)	0.0016 (9)
F2	0.073 (2)	0.0671 (17)	0.0510 (13)	−0.0439 (16)	0.0102 (13)	−0.0066 (12)
F3	0.0817 (16)	0.0321 (10)	0.100 (2)	−0.0239 (10)	−0.0659 (17)	0.0395 (14)
C10'	0.0500 (15)	0.0332 (13)	0.0339 (12)	−0.0136 (12)	−0.0226 (12)	0.0086 (11)
F1'	0.0424 (15)	0.0259 (10)	0.0397 (11)	−0.0088 (13)	−0.0189 (12)	0.0016 (9)
F2'	0.073 (2)	0.0671 (17)	0.0510 (13)	−0.0439 (16)	0.0102 (13)	−0.0066 (12)
F3'	0.0817 (16)	0.0321 (10)	0.100 (2)	−0.0239 (10)	−0.0659 (17)	0.0395 (14)
F4	0.0283 (7)	0.0378 (8)	0.0224 (6)	0.0013 (6)	0.0021 (5)	0.0022 (5)
F5	0.0321 (7)	0.0321 (7)	0.0269 (6)	0.0082 (6)	−0.0020 (6)	0.0041 (5)
F6	0.0436 (8)	0.0447 (8)	0.0222 (6)	−0.0058 (7)	−0.0129 (6)	−0.0034 (6)
O1	0.0443 (10)	0.0316 (9)	0.0205 (7)	0.0090 (7)	−0.0063 (7)	−0.0056 (7)
O2	0.0323 (8)	0.0267 (8)	0.0292 (8)	−0.0045 (7)	0.0027 (7)	0.0001 (7)
O3	0.0389 (9)	0.0275 (8)	0.0246 (8)	−0.0020 (7)	0.0071 (7)	−0.0027 (6)
N1	0.0260 (9)	0.0208 (9)	0.0237 (8)	−0.0023 (7)	−0.0064 (8)	0.0003 (7)
N2	0.0317 (10)	0.0225 (9)	0.0229 (9)	−0.0001 (8)	0.0029 (8)	0.0004 (8)
C1	0.0306 (11)	0.0230 (11)	0.0259 (10)	−0.0030 (9)	−0.0095 (9)	0.0006 (8)
C2	0.0312 (11)	0.0248 (11)	0.0250 (10)	−0.0013 (9)	−0.0091 (9)	−0.0005 (9)
C3	0.0246 (10)	0.0211 (10)	0.0199 (10)	0.0013 (9)	−0.0028 (8)	−0.0022 (8)
C4	0.0192 (9)	0.0193 (10)	0.0211 (9)	0.0024 (8)	0.0019 (8)	−0.0026 (8)
C5	0.0229 (10)	0.0228 (10)	0.0221 (9)	0.0001 (9)	0.0017 (8)	0.0006 (8)
C6	0.0255 (11)	0.0260 (11)	0.0304 (11)	−0.0058 (9)	0.0017 (9)	−0.0031 (9)
C7	0.0239 (10)	0.0284 (12)	0.0226 (9)	−0.0024 (9)	−0.0037 (8)	−0.0070 (9)
C8	0.0214 (10)	0.0257 (11)	0.0213 (10)	0.0012 (9)	−0.0008 (8)	−0.0035 (8)
C9	0.0184 (9)	0.0215 (10)	0.0207 (9)	0.0014 (8)	−0.0009 (8)	−0.0023 (8)
C11	0.0247 (10)	0.0300 (11)	0.0201 (10)	−0.0012 (9)	−0.0025 (8)	−0.0018 (8)
C12	0.0306 (11)	0.0220 (11)	0.0190 (9)	0.0014 (9)	−0.0022 (9)	−0.0012 (8)
C13	0.0298 (11)	0.0232 (12)	0.0223 (10)	−0.0005 (9)	−0.0036 (9)	−0.0004 (8)
C14	0.0389 (13)	0.0224 (11)	0.0326 (11)	0.0000 (10)	0.0024 (10)	0.0025 (9)
C15	0.0451 (14)	0.0284 (13)	0.0388 (13)	0.0025 (11)	0.0041 (11)	0.0102 (11)
C16	0.0454 (14)	0.0337 (14)	0.0298 (12)	0.0023 (12)	0.0025 (11)	0.0120 (10)
C17	0.0362 (12)	0.0341 (13)	0.0241 (10)	0.0065 (11)	−0.0047 (10)	0.0024 (9)
C18	0.0270 (10)	0.0238 (11)	0.0262 (10)	0.0025 (9)	−0.0001 (9)	−0.0009 (9)
C19	0.0257 (11)	0.0353 (13)	0.0233 (10)	0.0002 (10)	0.0035 (9)	−0.0002 (9)
C20	0.0407 (14)	0.0378 (14)	0.0305 (11)	0.0052 (11)	−0.0030 (11)	0.0020 (10)
C21	0.0269 (12)	0.0556 (17)	0.0365 (13)	0.0004 (12)	0.0037 (10)	−0.0108 (12)
C22	0.0416 (14)	0.0395 (14)	0.0344 (12)	−0.0026 (12)	0.0052 (11)	−0.0119 (11)

### Geometric parameters (Å, °)

**Table:**

C10—F3	1.319 (3)	C7—C8	1.361 (3)
C10—F1	1.338 (3)	C7—H7	0.9500
C10—F2	1.374 (3)	C8—C9	1.433 (3)
C10—C1	1.501 (3)	C8—C11	1.490 (3)
C10'—F1'	1.335 (5)	C12—C13	1.548 (3)
C10'—F2'	1.310 (5)	C12—H12	1.0000
C10'—C1	1.501 (3)	C13—C17	1.534 (3)
C10'—F3'	1.360 (5)	C13—H13	1.0000
F4—C11	1.352 (3)	C14—C15	1.526 (3)
F5—C11	1.347 (3)	C14—H14A	0.9900
F6—C11	1.344 (3)	C14—H14B	0.9900
O1—C12	1.425 (3)	C15—C16	1.515 (4)
O1—H1O	0.843 (11)	C15—H15A	0.9900
O2—C18	1.225 (3)	C15—H15B	0.9900
O3—C18	1.349 (3)	C16—C17	1.527 (4)
O3—C19	1.478 (3)	C16—H16A	0.9900
N1—C1	1.322 (3)	C16—H16B	0.9900
N1—C9	1.361 (3)	C17—H17A	0.9900
N2—C18	1.360 (3)	C17—H17B	0.9900
N2—C14	1.474 (3)	C19—C22	1.513 (4)
N2—C13	1.476 (3)	C19—C20	1.515 (4)
C1—C2	1.399 (3)	C19—C21	1.519 (4)
C2—C3	1.381 (3)	C20—H20A	0.9800
C2—H2	0.9500	C20—H20B	0.9800
C3—C4	1.421 (3)	C20—H20C	0.9800
C3—C12	1.517 (3)	C21—H21A	0.9800
C4—C9	1.422 (3)	C21—H21B	0.9800
C4—C5	1.425 (3)	C21—H21C	0.9800
C5—C6	1.364 (3)	C22—H22A	0.9800
C5—H5	0.9500	C22—H22B	0.9800
C6—C7	1.410 (3)	C22—H22C	0.9800
C6—H6	0.9500		
			
F3—C10—F1	107.2 (3)	C13—C12—H12	109.8
F3—C10—F2	106.9 (3)	N2—C13—C17	110.70 (18)
F1—C10—F2	104.3 (2)	N2—C13—C12	111.47 (18)
F3—C10—C1	114.1 (2)	C17—C13—C12	112.22 (19)
F1—C10—C1	112.6 (3)	N2—C13—H13	107.4
F2—C10—C1	111.2 (2)	C17—C13—H13	107.4
F1'—C10'—F2'	114.7 (11)	C12—C13—H13	107.4
F1'—C10'—C1	116.8 (12)	N2—C14—C15	111.9 (2)
F2'—C10'—C1	116.6 (6)	N2—C14—H14A	109.2
F1'—C10'—F3'	102.8 (10)	C15—C14—H14A	109.2
F2'—C10'—F3'	100.1 (8)	N2—C14—H14B	109.2
C1—C10'—F3'	102.1 (7)	C15—C14—H14B	109.2
C12—O1—H1O	111 (3)	H14A—C14—H14B	107.9
C18—O3—C19	121.93 (18)	C16—C15—C14	110.2 (2)
C1—N1—C9	116.55 (18)	C16—C15—H15A	109.6
C18—N2—C14	122.69 (18)	C14—C15—H15A	109.6
C18—N2—C13	118.66 (19)	C16—C15—H15B	109.6
C14—N2—C13	118.57 (17)	C14—C15—H15B	109.6
N1—C1—C2	125.2 (2)	H15A—C15—H15B	108.1
N1—C1—C10'	113.32 (18)	C15—C16—C17	109.9 (2)
C2—C1—C10'	121.47 (19)	C15—C16—H16A	109.7
N1—C1—C10	113.32 (18)	C17—C16—H16A	109.7
C2—C1—C10	121.47 (19)	C15—C16—H16B	109.7
C3—C2—C1	119.1 (2)	C17—C16—H16B	109.7
C3—C2—H2	120.5	H16A—C16—H16B	108.2
C1—C2—H2	120.5	C16—C17—C13	113.3 (2)
C2—C3—C4	118.04 (19)	C16—C17—H17A	108.9
C2—C3—C12	118.62 (19)	C13—C17—H17A	108.9
C4—C3—C12	123.32 (19)	C16—C17—H17B	108.9
C3—C4—C9	117.99 (19)	C13—C17—H17B	108.9
C3—C4—C5	123.76 (19)	H17A—C17—H17B	107.7
C9—C4—C5	118.23 (18)	O2—C18—O3	124.4 (2)
C6—C5—C4	121.0 (2)	O2—C18—N2	123.9 (2)
C6—C5—H5	119.5	O3—C18—N2	111.7 (2)
C4—C5—H5	119.5	O3—C19—C22	102.08 (19)
C5—C6—C7	120.8 (2)	O3—C19—C20	109.75 (19)
C5—C6—H6	119.6	C22—C19—C20	110.9 (2)
C7—C6—H6	119.6	O3—C19—C21	110.4 (2)
C8—C7—C6	120.2 (2)	C22—C19—C21	110.4 (2)
C8—C7—H7	119.9	C20—C19—C21	112.8 (2)
C6—C7—H7	119.9	C19—C20—H20A	109.5
C7—C8—C9	120.6 (2)	C19—C20—H20B	109.5
C7—C8—C11	119.99 (19)	H20A—C20—H20B	109.5
C9—C8—C11	119.39 (19)	C19—C20—H20C	109.5
N1—C9—C4	123.13 (18)	H20A—C20—H20C	109.5
N1—C9—C8	117.76 (18)	H20B—C20—H20C	109.5
C4—C9—C8	119.12 (19)	C19—C21—H21A	109.5
F6—C11—F5	105.93 (18)	C19—C21—H21B	109.5
F6—C11—F4	105.84 (18)	H21A—C21—H21B	109.5
F5—C11—F4	106.39 (18)	C19—C21—H21C	109.5
F6—C11—C8	112.43 (19)	H21A—C21—H21C	109.5
F5—C11—C8	112.38 (18)	H21B—C21—H21C	109.5
F4—C11—C8	113.30 (18)	C19—C22—H22A	109.5
O1—C12—C3	110.71 (18)	C19—C22—H22B	109.5
O1—C12—C13	105.59 (17)	H22A—C22—H22B	109.5
C3—C12—C13	110.95 (18)	C19—C22—H22C	109.5
O1—C12—H12	109.8	H22A—C22—H22C	109.5
C3—C12—H12	109.8	H22B—C22—H22C	109.5
			
C9—N1—C1—C2	−0.8 (3)	C3—C4—C9—C8	179.82 (19)
C9—N1—C1—C10'	−179.89 (19)	C5—C4—C9—C8	1.4 (3)
C9—N1—C1—C10	−179.89 (19)	C7—C8—C9—N1	178.1 (2)
F1'—C10'—C1—N1	170.2 (10)	C11—C8—C9—N1	−1.4 (3)
F2'—C10'—C1—N1	29.4 (10)	C7—C8—C9—C4	−2.1 (3)
F3'—C10'—C1—N1	−78.6 (7)	C11—C8—C9—C4	178.51 (19)
F1'—C10'—C1—C2	−8.9 (10)	C7—C8—C11—F6	6.8 (3)
F2'—C10'—C1—C2	−149.7 (10)	C9—C8—C11—F6	−173.75 (19)
F3'—C10'—C1—C2	102.3 (7)	C7—C8—C11—F5	−112.6 (2)
F1'—C10'—C1—C10	0(100)	C9—C8—C11—F5	66.8 (3)
F2'—C10'—C1—C10	0(98)	C7—C8—C11—F4	126.8 (2)
F3'—C10'—C1—C10	0(54)	C9—C8—C11—F4	−53.8 (3)
F3—C10—C1—N1	−51.6 (4)	C2—C3—C12—O1	−38.0 (3)
F1—C10—C1—N1	−174.0 (3)	C4—C3—C12—O1	143.6 (2)
F2—C10—C1—N1	69.4 (3)	C2—C3—C12—C13	78.9 (2)
F3—C10—C1—C2	129.3 (3)	C4—C3—C12—C13	−99.5 (2)
F1—C10—C1—C2	6.9 (4)	C18—N2—C13—C17	138.6 (2)
F2—C10—C1—C2	−109.7 (3)	C14—N2—C13—C17	−44.6 (3)
F3—C10—C1—C10'	0(16)	C18—N2—C13—C12	−95.8 (2)
F1—C10—C1—C10'	0(100)	C14—N2—C13—C12	81.1 (2)
F2—C10—C1—C10'	0(100)	O1—C12—C13—N2	179.09 (17)
N1—C1—C2—C3	−0.8 (4)	C3—C12—C13—N2	59.1 (2)
C10'—C1—C2—C3	178.2 (2)	O1—C12—C13—C17	−56.1 (2)
C10—C1—C2—C3	178.2 (2)	C3—C12—C13—C17	−176.08 (19)
C1—C2—C3—C4	1.9 (3)	C18—N2—C14—C15	−134.8 (2)
C1—C2—C3—C12	−176.6 (2)	C13—N2—C14—C15	48.5 (3)
C2—C3—C4—C9	−1.3 (3)	N2—C14—C15—C16	−53.7 (3)
C12—C3—C4—C9	177.02 (19)	C14—C15—C16—C17	58.2 (3)
C2—C3—C4—C5	177.0 (2)	C15—C16—C17—C13	−56.3 (3)
C12—C3—C4—C5	−4.7 (3)	N2—C13—C17—C16	47.6 (3)
C3—C4—C5—C6	−178.4 (2)	C12—C13—C17—C16	−77.6 (2)
C9—C4—C5—C6	−0.1 (3)	C19—O3—C18—O2	7.6 (3)
C4—C5—C6—C7	−0.6 (3)	C19—O3—C18—N2	−172.07 (19)
C5—C6—C7—C8	0.0 (3)	C14—N2—C18—O2	−179.1 (2)
C6—C7—C8—C9	1.3 (3)	C13—N2—C18—O2	−2.4 (3)
C6—C7—C8—C11	−179.2 (2)	C14—N2—C18—O3	0.6 (3)
C1—N1—C9—C4	1.4 (3)	C13—N2—C18—O3	177.28 (18)
C1—N1—C9—C8	−178.7 (2)	C18—O3—C19—C22	177.8 (2)
C3—C4—C9—N1	−0.3 (3)	C18—O3—C19—C20	60.1 (3)
C5—C4—C9—N1	−178.73 (19)	C18—O3—C19—C21	−64.8 (3)

### Hydrogen-bond geometry (Å, °)

**Table:**

*D*—H···*A*	*D*—H	H···*A*	*D*···*A*	*D*—H···*A*
O1—H1o···O2^i^	0.85 (3)	1.96 (3)	2.794 (3)	171 (3)

Symmetry codes: (i) *x*+1/2, −*y*+1/2, −*z*.
